# The Association of Kidney Function on Survival in Patients with Amyloid Light Chain Amyloidosis after Autologous Stem Cell Transplantation: A Multicenter Study

**DOI:** 10.34067/KID.0000001036

**Published:** 2025-11-10

**Authors:** Umut Selamet, Kole Joachim, Heather Lin, Gabriela Rondon, Charles S. Martinez, Ruby Delgado, Valda Page, Shveta S. Motwani, Berkay Dincer, Giada Bianchi, Muzaffar Qazilbash, Naoka Murakami, Ala Abudayyeh

**Affiliations:** 1 Department of Medical Oncology, Dana Farber Cancer Institute, Harvard Medical School, Boston, Massachusetts; 2 Division of Renal Medicine, Department of Medicine, Brigham and Women's Hospital, Harvard Medical School, Boston, Massachusetts, USA; 3 Department of Biostatistics, The University of Texas MD Anderson Cancer Center, Houston, Texas; 4 Department of Stem Cell Transplantation and Cellular Therapy, The University of Texas MD Anderson Cancer Center, Houston, Texas; 5 Division of Internal Medicine, Section of Nephrology, The University of Texas MD Anderson Cancer Center, Houston, Texas; 6 Division of Hematology, Department of Medicine, Brigham and Women's Hospital, Harvard Medical School Boston, Massachusetts

**Keywords:** CKD, onco-nephrology, renal failure, survival, survival analysis

## Abstract

**Key Points:**

- Almyloid light chain amyloid patients post autologous stem cell transplantation with higher eGFR was not associated with better progression-free survival but was associated with increased overall survival (OS).
- Patients with kidney amyloidosis stage 3 at baseline had inferior OS compared with those who had stage 1.
- OS was better in those who had complete kidney response irrespective of the baseline kidney amyloidosis stage, compared with those without response.

**Background:**

We investigated how kidney function affected progression-free survival (PFS) and overall survival (OS) after autologous stem cell transplantation (ASCT) in patients with amyloid light chain (AL) amyloidosis.

**Methods:**

We performed a retrospective cohort study of 314 patients with AL amyloidosis who underwent ASCT between 2010 and 2020. In addition to the baseline demographics, comorbidities, and kidney amyloidosis stage, eGFR and other laboratory values were collected at day 0, day 100, 6 months, 1 year, 2 years, and 3 years after ASCT. The Cox proportional hazards models were used to estimate hazard ratio (HR) based on landmark analysis for the effects of eGFR and other longitudinal measures on PFS or OS.

**Results:**

Higher eGFR values at all time points after ASCT were not significantly associated with longer PFS but was associated with increased OS. Patients with kidney amyloidosis stage 3 at baseline had inferior OS compared with those who had stage 1 (HR [95% confidence interval] = 9.428 [1.134 to 78.381], *P* value = 0.0379). OS was better in those who had complete kidney response irrespective of the baseline kidney amyloidosis stage, compared with those without response (HR [95% confidence interval] = 7.581 [2.042 to 28.149], *P* = 0.0025).

**Conclusions:**

This retrospective study shows that kidney function has an effect on survival in patients with AL amyloidosis who undergo ASCT. Both higher eGFR at baseline and complete kidney response after ASCT favor improved survival outcomes.

## Visual Abstract

**Figure:**
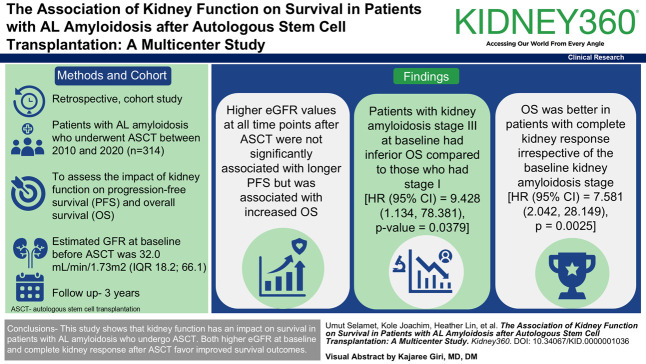

## Introduction

Immunoglobin light chain (amyloid light chain [AL]) amyloidosis is a life-threatening clonal plasma cell disorder that is characterized by extracellular deposition of abnormal protein aggregates, known as amyloid fibrils, within various organs and tissues. While amyloidosis can affect any organ, the involvement of the kidneys is particularly common, affecting more than two thirds of such patients, and leading to progressive kidney dysfunction.^1^ This is typically manifested by proteinuria and progressive CKD often resulting in the need for KRT. Treatment of the underlying plasma cell dyscrasia has been shown to improve kidney AL amyloidosis mainly due to the hematologic response.^2–4^

Autologous stem cell transplantation (ASCT) after high dose melphalan therapy has been used as a therapeutic option in many patients in the United States^5^ and has led to improved survival^6^ among eligible patients. Outcomes data among patients with AL amyloidosis and impaired kidney function who undergo ASCT are limited. In one retrospective study, post-ASCT outcomes were compared between patients with eGFR <45 and ≥45 ml/min.^7^ Those with eGFR <45 ml/min had higher rates of hospitalization, progression to dialysis, and early mortality. Other studies indicated that improved survival of AL amyloidosis patients after ASCT was associated with hematologic response to treatment but long-term data about predictors of survival are needed.^5,8,9^ We assembled a large multicenter cohort of patients with AL amyloidosis who were treated with high dose melphalan with a primary aim of identifying factors associated with eGFR change over time and secondary aim to assess the effect of kidney function on progression-free survival (PFS) and overall survival (OS).

## Methods

### Study Design and Patient Population

This retrospective study was approved by the Institutional Review Board at the University of Texas MD Anderson Cancer Center, Dana Farber Cancer Institute, and Mass General Brigham in accordance with the principles of the declaration of Helsinki. Patients were identified by querying the MD Anderson and Mass General Brigham database for patients with AL amyloidosis who underwent ASCT between January 1, 2010, and December 31, 2020. Data sharing was conducted under a formal Medical Data Transfer Agreement between institutions.

### Data Collection and Definitions

Detailed demographic information for each patient, including age, sex, and race and ethnicity (Asian, Black, Hispanic, and White) were collected from the database. Data on cumulative melphalan dose for conditioning at the time of ASCT were captured. Data regarding comorbidities, individual organ involvement, and cytogenetics were collected.

We also collected data on serum creatinine, eGFR calculated by using the CKD Epidemiology Collaboration equation,^8^ serum calcium, albumin, lactate dehydrogenase, hemoglobin, *N*-terminal pro b-type natriuretic peptide, serum kappa and lambda light chain levels and spot urine total protein, spot urine creatinine, and/or 24-hour urine protein levels at days 0, 100, month 6, and years 1, 2, and 3 after ASCT.

For hematologic response assessment of amyloidosis (complete response [CR]; partial response [PR]; very good partial response [VGPR]; stable disease; progressive disease) after ASCT, the Center for International Blood and Marrow Transplant Research and International Myeloma Working Group guidelines were used before 2020 based on Palladini *et al.*, and thereafter, the National Comprehensive Cancer Network guidelines were used.^9,10^

Amyloidosis relapse was defined as the disease either ceasing to respond to therapy or progressing within 60 days after the last treatment after having shown at least a minimal response initially (*i.e*., 25%–49% reduction in serum monoclonal paraprotein or plasma cells in bone marrow for at least 6 weeks or 50%–89% reduction in 24-hour urinary light chain excretion, which still exceeds 200 mg/24 hours, for at least 6 weeks, with no increase in the size or number of lytic bone lesions). Primary refractory amyloidosis was defined as nonresponsive to treatment, that is, disease that has never shown a minimal response or improvement with any therapy.^11^

We used the criteria first described by Palladini *et al.* for staging of kidney amyloidosis for patients with kidney involvement of AL amyloidosis.^2^ Stage 1 was defined as both eGFR >50 ml/min per 1.73 m^2^ and proteinuria <5 g/24 h, stage 3 was defined as both eGFR <50 ml/min per 1.73 m^2^ and proteinuria >5 g/24 h, and stage 2 was defined as either eGFR <50 ml/min per 1.73 m^2^ or proteinuria >5 g/24 h (Supplemental Table 1).

We used graded kidney response criteria described by Muchtar *et al*.^12^ for patients with kidney involvement of AL amyloidosis (Supplemental Table 2).

The data supporting this study's findings are available from the corresponding authors on request.

### Statistical Analysis

Given the longitudinal nature of the data, linear mixed effect models were used to study the change of eGFR over time to take the intrapatient correlation into account.^13^ We first evaluated the association of eGFR with each of the covariates in a model including indicators of time points and the covariate. We then checked time by the covariate interaction on eGFR level. Finally, a linear mixed effect model was developed with all of the covariates and interaction terms with a *P* value of ≤ 0.05 in the aforementioned steps. Only the terms with a *P* value of 0.05 or less remained in the final model. When an interaction term was significant at 0.05 significance level, the related main effect terms were kept in the model regardless of whether they were significant. The continuous variables of eGFR, albumin, hemoglobin, and lactate dehydrogenase were logarithmically transformed to the base of two to achieve a more symmetric distribution and to reduce the influence of potential outliers.

The distributions of PFS and OS were estimated by the Kaplan-Meier method. We then assessed associations of eGFR with PFS and OS. PFS was defined as the duration from the date of ASCT to the date of progression or death, whichever occurred first. OS was defined as the duration from the date of ASCT to the date of death. For events that had not occurred by the time of data analysis, times were censored at the last contact at which the patient was known to be progression free for PFS or alive for OS. The log-rank test was performed to test the difference in survival between groups.^14^ Regression analyses of survival data based on the Cox proportional hazards model were conducted on PFS and OS.^15^ We performed landmark analysis to evaluate the effects of eGFR on PFS or OS on days 0, 100, month 6, and years 1, 2, and 3 post-ASCT. We used SAS version 9.4, R version 4.2.0, and S-Plus version 8.2 for statistical analyses.^16^

## Results

### Patient Characteristics

Patient characteristics are summarized in Table 1. We collected 314 consecutive cases of AL amyloidosis in MD Anderson Cancer Center, Dana Farber Cancer Institute and Brigham and Women's Hospital from 2010 to 2020. The median age at the time of ASCT was 61.3 years (interquartile range [IQR], 53.8–67.0), and 63.1% were male. Most (66.9%) patients were White, followed by Black (15.3%) and Hispanic (12.1%) race and ethnicity. Amyloid involvement of the heart, kidney, and gastrointestinal tract were reported in 22.9%, 34.4%, and 7.3%, respectively. Lambda predominant amyloidosis was slightly more common (56.0%) than kappa (44.0%) predominant amyloidosis. eGFR at baseline before ASCT was 32.0 ml/min per 1.73 m^2^ (IQR, 18.2–66.1) with a median proteinuria of 3.0 g/24 h or g/gCr (IQR, 0.5–6.8). A significant proportion (43.6%) achieved PR before ASCT, followed by stable disease (14.1%). For conditioning regimen, 99% received melphalan, of which 42.4% received high dose (>200 mg/m^2^).

**Table 1:** Patients characteristics

Variable	Category	Frequency count (Total *n*=314)	%
Sex	Female	116	36.94
	Male	198	63.06
Race	Asian	10	3.18
	Black	48	15.29
	Hispanic	38	12.10
	Missing	1	0.32
	Other	7	2.23
	White	210	66.88
Response before ASCT	Missing	7	2.23
	CR	29	9.24
	PD	23	7.32
	PR	137	43.63
	SD	44	14.01
	VGPR	74	23.57
Amyloidosis: Heart	No	242	77.07
	Yes	72	22.93
Amyloidosis: Kidney	No	206	65.61
	Yes	108	34.39
Amyloidosis: GI	No	291	92.68
	Yes	23	7.32
Light chain	Kappa	138	43.95
	Lambda	176	56.05
Prep-regimen	BEAM	1	0.32
	Gemcitabine/Busulfan/Melphalan/Panobinostat	1	0.32
	Melphalan	311	99.05
	Melphalan/Revlimid	1	0.32
Standard dose	No	181	57.64
	Yes	133	42.36

ASCT, autologous stem cell transplantation; BEAM, BCNU (Carmustine), Etoposide, Ara-C (Cytarabine), and Melphalan; CR, complete response; GI, gastrointestinal; PD, progressive disease; PR, partial response; Prep-regimen, preparatory regimen; SD, stable disease; VGPR, very good partial response.

### Overall Hematologic Outcomes Post-ASCT

Post-ASCT hematologic response was overall favorable: 50% achieved CR, 28.7% achieved VGPR, and 16.6% achieved PR. Although 40.8% experienced progression during the follow-up period, 36.3% and 12.4% maintained CR and VGPR, respectively.

### Associations between Patient Factors and eGFR Over Time

We investigated the associations between eGFR and patients' clinical factors at different time points and included the results in Table 2. On bivariate analysis, starting from 6 months post-ASCT, eGFR declined over time (*P* < 0.0001). Black and Hispanic races were associated with lower eGFR (*P* < 0.0075). Amyloid involvement of heart, kidney, or gastrointestinal system were associated with lower eGFR (*P* ≤ 0.001). Older patients had lower eGFR (*P* = 0.0286). Patients with cerebrovascular, cardiac, and/or lung disease had lower eGFR (*P* < 0.0346). Obese patients had lower eGFR (*P* = 0.0269). Patients with higher *N*-terminal pro b-type natriuretic peptide levels had lower eGFR (*P* < 0.0001). The eGFR was higher for every fold increase in hemoglobin and lower for every fold increase in serum light chains (*P* < 0.0001). Higher melphalan dose was associated with higher eGFR (*P* < 0.0001).

**Table 2:** Univariate and multivariable analysis of eGFR using preautologous stem cell transplantation patients' characteristics

Model	Variable	Levels	Univariable				Multivariable			
			Estimate	Standard error	Pr > |t|	Pr > F	Estimate	Standard error	Pr > |t|	Pr > F
2	Gender	Female	0.06382	0.1333	0.6324	0.6324				
		Male	0							
	Time	D 100	0.03870	0.03576	0.2800	0.0002				
		Mo 6	−0.05823	0.02430	0.0172					
		Yr 1	−0.08920	0.02780	0.0015					
		Yr 2	−0.1319	0.05093	0.0102					
		Yr 3	−0.1705	0.05211	0.0013					
		Baseline	0							
		Baseline	0							
3	Light chains	Kappa	−0.6053	0.1251	<0.0001	<0.0001	−0.2760	0.1095	0.0123	0.0123
		Lambda	0				0			
	Time	D 100	0.03770	0.03573	0.2922	0.0002				
		Mo 6	−0.05842	0.02430	0.0169					
		Yr 1	−0.08910	0.02779	0.0015					
		Yr 2	−0.1338	0.05098	0.0092					
		Yr 3	−0.1734	0.05217	0.0011					
		Baseline	0							
4	Disease response at baseline	PD	−1.0391	0.3112	0.0009	0.0019	−0.6787	0.2529	0.0077	0.0461
		PR	−0.05684	0.2272	0.8026		−0.2040	0.1866	0.2753	
		SD	0.06144	0.2656	0.8173		−0.02641	0.2166	0.9030	
		VGPR	−0.1224	0.2435	0.6157		−0.1130	0.1986	0.5699	
		CR	0				0			
	Time	D 100	0.03480	0.03659	0.3422	0.0004				
		Mo 6	−0.06019	0.02483	0.0160					
		Yr 1	−0.08777	0.02826	0.0021					
		Yr 2	−0.1127	0.04911	0.0226					
		Yr 3	−0.1625	0.05303	0.0026					
		Baseline	0							
5	Race	Black	−0.8096	0.3135	0.0103	0.0006	−0.5723	0.2588	0.0278	0.0023
		Hispanic	−0.7307	0.3238	0.0247		−0.6441	0.2685	0.0171	
		Others	0				0			
		White	−0.2054	0.2802	0.4640		−0.1866	0.2342	0.4263	
	Time	D 100	0.04039	0.03590	0.2615	0.0002				
		Mo 6	−0.05808	0.02438	0.0179					
		Yr 1	−0.08763	0.02789	0.0019					
		Yr 2	−0.1294	0.05110	0.0120					
		Yr 3	−0.1663	0.05217	0.0017					
		Baseline	0							
6	GI amyloidosis	Yes versus No	0.7976	0.2422	0.0011	0.0011				
	Time	D 100	0.03819	0.03577	0.2865	0.0002				
		Mo 6	−0.05835	0.02431	0.0170					
		Yr 1	−0.08958	0.02782	0.0014					
		Yr 2	−0.1328	0.05095	0.0097					
		Yr 3	−0.1723	0.05217	0.0012					
		Baseline	0							
7	Cardiac amyloidosis	Yes versus No	0.8578	0.1449	<0.0001	<0.0001	0.4884	0.1296	0.0002	0.0002
	Time	D 100	0.03828	0.03573	0.2850	0.0002				
		Mo 6	−0.05813	0.02430	0.0174					
		Yr 1	−0.08894	0.02779	0.0015					
		Yr 2	−0.1322	0.05097	0.0101					
		Yr 3	−0.1721	0.05211	0.0012					
		Baseline	0							
8	Kidney amyloidosis	Yes vs no	0.7516	0.1288	<0.0001	<0.0001	0.3463	0.1178	0.0036	0.0036
	Time	D 100	0.03749	0.03576	0.2954	0.0001				
		Mo 6	−0.05837	0.02431	0.0170					
		Yr 1	−0.08929	0.02780	0.0015					
		Yr 2	−0.1370	0.05100	0.0077					
		Yr 3	−0.1769	0.05225	0.0009					
		Baseline	0							
9	Age at ASCT	Per fold/unit increase	−0.01499	0.006814	0.0286	0.0286				
	Time	D 100	0.03871	0.03576	0.2798	0.0002				
		Mo 6	−0.05811	0.02430	0.0175					
		Yr 1	−0.08938	0.02780	0.0015					
		Yr 2	−0.1318	0.05093	0.0102					
		Yr 3	−0.1703	0.05204	0.0013					
		Baseline	0							
10	Cerebrovascular disease	Yes versus No	0.8986	0.2982	0.0028	0.0028				
	Time	D 100	0.03787	0.03585	0.2916	0.0002				
		Mo 6	−0.05975	0.02434	0.0147					
		Yr 1	−0.09042	0.02786	0.0013					
		Yr 2	−0.1313	0.05116	0.0109					
		Yr 3	−0.1704	0.05228	0.0014					
		Baseline	0							
11	Diabetes	Yes versus No	0.008576	0.1772	0.9614	0.9614				
	Time	D 100	0.03866	0.03576	0.2805	0.0002				
		Mo 6	−0.05823	0.02430	0.0172					
		Yr 1	−0.08922	0.02780	0.0015					
		Yr 2	−0.1319	0.05094	0.0102					
		Yr 3	−0.1704	0.05211	0.0013					
		Baseline	0							
12	Liver disease	Yes versus No	−0.03060	0.3036	0.9198	0.9198				
	Time	D 100	0.03864	0.03576	0.2808	0.0002				
		Mo 6	−0.05824	0.02430	0.0172					
		Yr 1	−0.08923	0.02780	0.0015					
		Yr 2	−0.1320	0.05094	0.0102					
		Yr 3	−0.1704	0.05211	0.0013					
		Baseline	0							
13	Log albumin	Per fold increase	0.007282	0.05364	0.8920	0.8920				
	Time	D 100	0.03342	0.03885	0.3902	0.0003				
		Mo 6	−0.06565	0.02854	0.0219					
		Yr 1	−0.08916	0.03166	0.0051					
		Yr 2	−0.1267	0.04768	0.0083					
		Yr 3	−0.1688	0.05140	0.0012					
		Baseline	0							
14	Log hemoglobin	Per fold increase	0.3695	0.06188	<0.0001	<0.0001	0.2763	0.06252	<0.0001	<0.0001
	Time	D 100	−0.01713	0.03756	0.6486	<0.0001				
		Mo 6	−0.1285	0.02703	<0.0001					
		Yr 1	−0.1665	0.02932	<0.0001					
		Yr 2	−0.2101	0.04622	<0.0001					
		Yr 3	−0.2465	0.04804	<0.0001					
		Baseline	0							
15	Log_Kappa–lambda	Per fold increase	−0.05789	0.007490	<0.0001	<0.0001	−0.04896	0.007538	<0.0001	<0.0001
	Time	D 100	−0.04709	0.03568	0.1878	<0.0001				
		Mo 6	−0.1341	0.02584	<0.0001					
		Yr 1	−0.1219	0.02659	<0.0001					
		Yr 2	−0.1498	0.04376	0.0007					
		Yr 3	−0.1844	0.04686	0.0001					
		Baseline	0							
16	Log LDH	Per fold increase	−0.02186	0.02469	0.3762	0.3762				
	Time	D 100	0.03395	0.03746	0.3655	0.0003				
		Mo 6	−0.07157	0.02567	0.0057					
		Yr 1	−0.08392	0.02869	0.0038					
		Yr 2	−0.1117	0.04706	0.0185					
		Yr 3	−0.1605	0.05092	0.0019					
		Baseline	0							
17	Log NT-ProBNP	Per fold increase	−0.09746	0.01604	<0.0001	<0.0001				
	Time	D 100	0.003801	0.05250	0.9425	0.0001				
		Mo 6	−0.06286	0.03994	0.1202					
		Yr 1	0.04746	0.06050	0.4367					
		Yr 2	−0.1605	0.06195	0.0115					
		Yr 3	−0.3044	0.06443	<0.0001					
		Baseline	0							
18	Obesity	Yes versus No	−0.4307	0.1937	0.0269	0.0269				
	Time	D 100	0.03857	0.03577	0.2817	0.0002				
		Mo 6	−0.05830	0.02431	0.0171					
		Yr 1	−0.08936	0.02781	0.0015					
		Yr 2	−0.1316	0.05095	0.0104					
		Yr 3	−0.1700	0.05208	0.0013					
		Baseline	0							
19	Cardiac disease	Yes versus No	−0.3941	0.1857	0.0346	0.0346				
	Time	D 100	0.03879	0.03578	0.2792	0.0002				
		Mo 6	−0.05837	0.02431	0.0170					
		Yr 1	−0.08924	0.02779	0.0015					
		Yr 2	−0.1302	0.05089	0.0111					
		Yr 3	−0.1681	0.05200	0.0015					
		Baseline	0							
20	Lung disease	Yes versus No	−0.3419	0.1278	0.0079	0.0079				
	Time	D 100	0.03761	0.03577	0.2938	0.0002				
		Mo 6	−0.05850	0.02430	0.0167					
		Yr 1	−0.08963	0.02780	0.0014					
		Yr 2	−0.1333	0.05094	0.0095					
		Yr 3	−0.1721	0.05210	0.0012					
		Baseline	0							
21	Melphalan dose	≥200 versus<200	0.8994	0.1198	<0.0001	<0.0001	0.6964	0.1080	<0.0001	<0.0001
	Time	D 100	0.03764	0.03577	0.2935	0.0002				
		Mo 6	−0.05776	0.02428	0.0180					
		Yr 1	−0.08872	0.02779	0.0016					
		Yr 2	−0.1323	0.05084	0.0098					
		Yr 3	−0.1716	0.05174	0.0011					
		Baseline	0							

ASCT, autologous stem cell transplantation; CR, complete response; GI, gastrointestinal; LDH, lactate dehydrogenase; NT-ProBNP, *N*-terminal pro b-type natriuretic peptide; PD, progressive disease; PR, partial response; SD, stable disease; VGPR, very good partial response.

In a multivariate analysis (Table 2), race was significantly associated with eGFR (*P* = 0.00752). Specifically, compared with the patients with other race, Black and Hispanic patients had lower eGFR (*P* = 0.0023). Patients with a progressive disease at baseline had lower eGFR than those with a CR (*P* = 0.046). Patients with amyloid involvement of heart and kidney had lower eGFR (*P* = 0.0002 and 0.0036, respectively).

Patients treated with melphalan doses of >200 mg/m^2^ had higher eGFR (0.62-fold higher, *P* < 0.0001). Patients with higher hemoglobin levels had higher eGFR (increase by 0.27-fold per folder increase, *P* < 0.0001). Patients with higher serum kappa or lambda levels had lower eGFR (decrease by 0.048-fold per folder increase, *P* < 0.0123). None of the interaction terms between patient characteristic and time were included in the final multivariate model (*P* > 0.05).

### Association of eGFR and Other Variables on PFS

**Figure 1:**
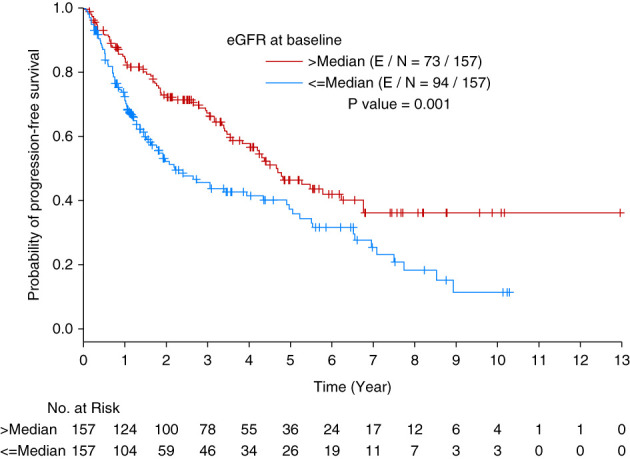
**Kaplan-Meier curve of PFS by eGFR at baseline.** PFS, progression-free survival.

Using landmark analysis for all time points (baseline, 100 days, 6 months, 1 year, 2 years, and 3 years), we evaluated the effect of eGFR on PFS. The median PFS for the entire population was 3.94 years. In a multivariable analysis, after adjusting for the other factors, eGFR at 100 days was not significantly associated with PFS (hazard ratio [HR]=1.336; [95% confidence interval [CI] 0.924 to 1.929] for ≤median vs >median, *P* = 0.1233 Figure 1 and Table 3). In multivariable analysis, the other factors that were associated with PFS at different time points were also evaluated higher (> median) serum light chain level at all time points was associated with poor PFS (*P* ≤ 0.0142, Tables 3 and 4). Kidney amyloid involvement was independently associated with poor PFS at baseline, 100 days, 6 months, and 1 year (*P* < 0.0094, Tables 3 and 4). Treating patients with higher doses of melphalan (>200 mg/m^2^) was associated with better PFS at baseline, 100 days, and 6 months (*P* < 0.0066, Table 3). Higher serum albumin level was associated with improved PFS at 3 years post-ASCT (*P* = 0.013, Table 4). We also evaluated the continuous variables (*e.g*., eGFR, Kappa/Lambda, albumin, hemoglobin *etc.*) in relation to PFS and OS using quartiles, and the conclusions remained unchanged (see Supplemental Tables 3 and 4).

**Table 3:** Associations of progression-free survival with patients' clinical, tumor characteristics, and baseline laboratory measures in multivariable setting at baseline, 100 days, and 6 months

Parameter	Level	Baseline			100 d			6 mo		
		HR (95% CI)	*P* ^a^	*P* ^b^	HR (95% CI)	*P* ^a^	*P* ^b^	HR (95% CI)	*P* ^a^	*P* ^b^
Kidney amyloidosis	Yes vs no	0.599 (0.41 to 0.875)	0.0081	0.0081	0.591 (0.398 to 0.879)	0.0094	0.0094	0.579 (0.383 to 0.875)	0.0095	0.0095
Melphalan dose	≥200 mg/m^2^ vs<200 mg/m^2^	0.678 (0.48 to 0.959)	0.0278	0.0278	0.668 (0.465 to 0.96)	0.0290	0.0290	0.594 (0.408 to 0.865)	0.0066	0.0066
eGFR	≤median vs >median	1.147 (0.793 to 1.66)	0.4653	0.4653	1.336 (0.924 to 1.929)	0.1233	0.1233	1.03 (0.697 to 1.522)	0.8818	0.8818
Kappa–lambda	≤median vs >median	0.529 (0.373 to 0.751)	0.0004	0.0004	0.644 (0.453 to 0.915)	0.0142	0.0142	0.611 (0.417 to 0.895)	0.0115	0.0115

CI, confidence interval; HR, hazard ratio.

a *P* value for comparison with reference group.

b *P* value for overall effect.

**Table 4:** Associations of progression-free survival with patients' clinical, tumor characteristics, and baseline laboratory measures in multivariable setting at 1 year, 2 years, and 3 years

Parameter	Level	1 yr			2 yr			3 yr		
		HR (95% CI)	*P* ^a^	*P* ^b^	HR (95% CI)	*P* ^a^	*P* ^b^	HR (95% CI)	*P* ^a^	*P* ^b^
Kidney amyloidosis	Yes vs no	0.542 (0.341 to 0.86)	0.0094	0.0094						
Albumin	≤median vs >median							2.716 (1.235 to 5.973)	0.0130	0.0130
eGFR	≤median vs >median	0.936 (0.588 to 1.49)	0.7800	0.7800	0.989 (0.542 to 1.802)	0.9702	0.9702	0.634 (0.308 to 1.308)	0.2179	0.2179
Kappa–lambda	≤median vs >median	0.412 (0.259 to 0.654)	0.0002	0.0002	0.364 (0.196 to 0.678)	0.0014	0.0014	0.285 (0.133 to 0.611)	0.0012	0.0012

CI, confidence interval; HR, hazard ratio.

a *P* value for comparison with reference group.

b *P* value for overall effect.

### Associations of eGFR and Other Variables with OS

**Figure 2:**
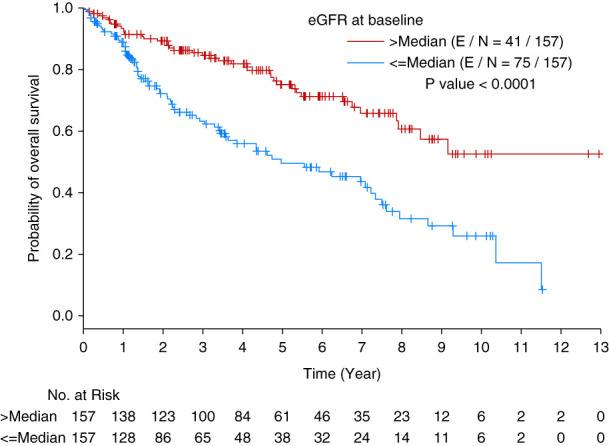
**Kaplan-Meier curve of OS by eGFR at baseline.** OS, overall survival.

Using landmark analysis for all time points (baseline, 100 days, 6 months, 1 year, 2 years, and 3 years), we evaluated the effect of eGFR on OS. The median OS for the entire population was 7.87 years. In a multivariable analysis, eGFR was significantly associated with OS after adjusting for the other variables at baseline (HR=2.326 [95% CI, 1.586 to 3.412] for ≤median vs >median, *P* < 0.0001 [Figure 2]), as well as at 100 days, 6 months, 2 years, and 3 years (Tables 5 and 6). Regarding age at the time of ASCT, for every year increase, there was an associated decreased survival at all time points except at 3 years (*P*≤0.0039, Tables 5 and 6). Every log increase in serum light chain levels was associated with poor survival at 100 days, 6 months, 1 year, and 2 years post-ASCT (Tables 5 and 6). Kidney amyloid involvement was independently associated with poor survival at 1-year post-ASCT (*P* = 0.019, Table 6). Receiving >200 mg/m^2^ of melphalan was associated with improved OS at 1 year (*P* = 0.042, Table 6). At 1 year and 3 years, higher hemoglobin was associated with improved survival (*P* ≤ 0.031, Table 6).

**Table 5:** Associations of overall survival with patients' clinical, tumor characteristics, and laboratory measures at baseline, 100 days, and 6 months in multivariable settings

Parameter	Level	Baseline			100 d			6 mo		
		HR (95% CI)	*P* ^a^	*P* ^b^	HR (95% CI)	*P* ^a^	*P* ^b^	HR (95% CI)	*P* ^a^	*P* ^b^
Age at transplant	Per year increase	1.041 (1.018 to 1.063)	0.0003	0.0003	1.045 (1.019 to 1.071)	0.0005	0.0005	1.045 (1.02 to 1.07)	0.0003	0.0003
Kidney amyloidosis	Yes vs no									
Melphalan dose	≥200 vs<200							0.608 (0.381 to 0.972)	0.0375	0.0375
eGFR	≤median vs >median	2.326 (1.586 to 3.412)	<0.0001	<0.0001	2.322 (1.49 to 3.618)	0.0002	0.0002	2.091 (1.32 to 3.313)	0.0017	0.0017
Kappa/Lambda	≤median vs >median				0.578 (0.373 to 0.894)	0.0137	0.0137	0.613 (0.395 to 0.951)	0.0291	0.0291

CI, confidence interval; HR, hazard ratio.

a *P* value for comparison with reference group.

b *P* value for overall effect.

**Table 6:** Associations of overall survival with patients' clinical, tumor characteristics, and laboratory measures at 1 year, 2 years, and 3 years in multivariable settings

Parameter	Level	1 yr			2 yr			3 yr		
		HR (95% CI)	*P* ^a^	*P* ^b^	HR (95% CI)	*P* ^a^	*P* ^b^	HR (95% CI)	*P* ^a^	*P* ^b^
Age at transplant	Per year increase	1.037 (1.012 to 1.063)	0.0039	0.0039	1.056 (1.022 to 1.092)	0.0012	0.0012			
Kidney amyloidosis	Yes vs no	0.526 (0.308 to 0.9)	0.0190	0.0190				0.403 (0.19 to 0.857)	0.0182	0.0182
Melphalan dose	≥200 vs<200	0.588 (0.353 to 0.982)	0.0424	0.0424						
eGFR	≤median vs >median	1.345 (0.777 to 2.328)	0.2903	0.2903	2.332 (1.239 to 4.387)	0.0086	0.0086	1.954 (0.942 to 4.051)	0.0718	0.0718
Kappa–lambda	≤median vs >median	0.452 (0.263 to 0.777)	0.0041	0.0041	0.381 (0.194 to 0.748)	0.0051	0.0051			
Hemoglobin	≤median vs >median	1.785 (1.054 to 3.021)	0.0310	0.0310				2.968 (1.363 to 6.464)	0.0062	0.0062

CI, confidence interval; HR, hazard ratio.

a *P* value for comparison with reference group.

b *P* value for overall effect.

### Association of Kidney Amyloidosis Stage with PFS and OS

**Figure 3:**
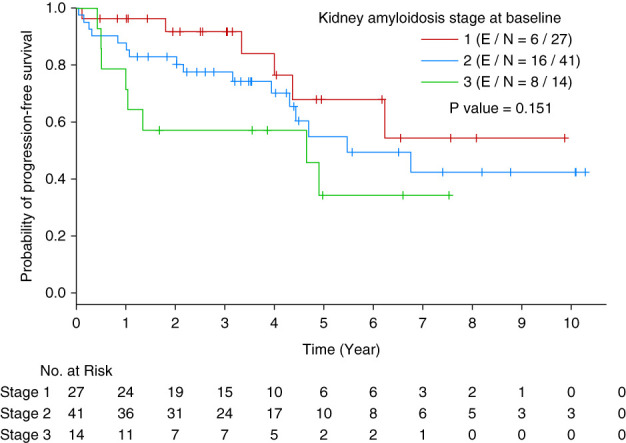
Kaplan-Meier curve of PFS by kidney amyloidosis stage at baseline.

One hundred and eight patients had kidney involvement of AL amyloidosis (*n*=108 patients) at the time of ASCT. Kidney amyloidosis stage 1 was observed in 27 patients, stage 2 in 41 patients, stage 3 in 14 patients, and unknown stage due to missing data in 26 patients. Since Palladini's kidney amyloidosis staging has been validated only for baseline, we used kidney amyloidosis stage at baseline in analysis. At baseline, there were no associations between advanced kidney amyloidosis stage and worse PFS (*P* ≥ 0.151, Figure 3).

**Figure 4:**
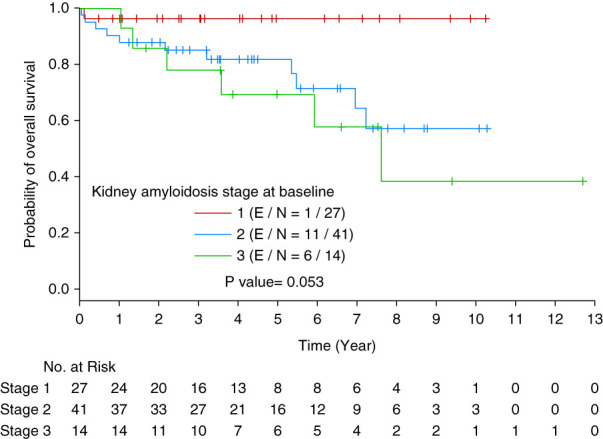
Kaplan-Meier curve of OS by kidney amyloidosis stage at baseline.

However, when we evaluated kidney amyloidosis stage and OS, we observed that patients who had stage 3 kidney amyloidosis at baseline had inferior OS compared with those who had stage 1 kidney amyloidosis (HR = 9.428 [95% CI, 1.134 to 78.381], *P* value = 0.05, Figure 4).

### Association of Kidney Response at 6 months with PFS

**Figure 5:**
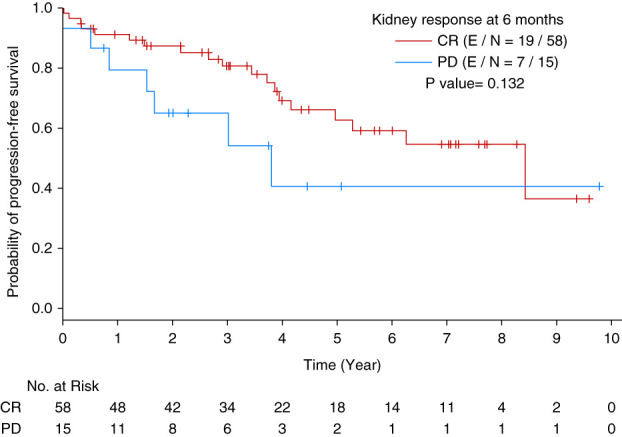
**Kaplan-Meier curve of PFS by kidney response 6 months post-ASCT.** ASCT, autologous stem cell transplantation; CR, complete response; PD, progressive disease.

In 108 patients who had kidney involvement of AL amyloidosis, kidney response was evaluated only at month 6 post-ASCT due to missing data at farther time points. At month 6, 60 patients (55%) had complete kidney response, 18 patients (17%) had progressive kidney disease, and 30 patients (28%) could not be assessed due to missing data. PFS did not significantly differ between patients with kidney progression and patients with complete kidney response at month 6 (*P* = 0.132, Figure 5). After adjusting for baseline kidney amyloidosis stage, PFS still did not significantly differ between patients with kidney progression and patients with complete kidney response at month 6 (HR = 2.568 [95% CI, 0.875 to 7.541], *P* = 0.0861).

**Figure 6:**
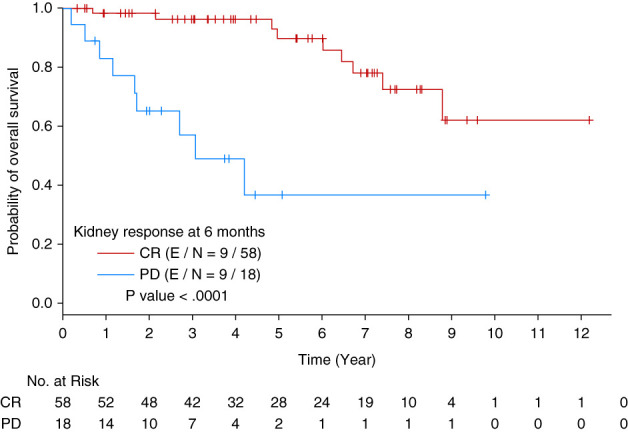
Kaplan-Meier curve of OS by kidney response 6 months post-ASCT.

Patients who had kidney progression at month 6 had inferior OS than those who had CR when unadjusted for baseline kidney amyloidosis stage (HR = 7.012 [95% CI, 2.635 to 18.664], *P* < 0.0001, Figure 6). After adjustment for baseline kidney amyloidosis stage, patients with kidney progression at month 6 still had inferior OS than those who had complete kidney response (HR = 7.581 [95% CI, 2.042 to 28.149], *P*=0.0025). However, the difference at OS between the patients with stage 3 baseline kidney amyloidosis and those with stage 1 was insignificant (*P* = 0.1866).

## Discussion

In this study, we evaluated the factors associated with eGFR overtime in patients with AL amyloidosis and undergone ASCT. The effect of eGFR and other disease-related variables were also evaluated in relation to PFS and OS. We found that kidney involvement and response were important predictors of PFS and OS after ASCT.

In line with several previous studies, our study demonstrated that higher dose of melphalan (>200 mg/m^2^) conditioning for ASCT was associated with improved eGFR, PFS, and OS.^3,6,17^ In addition, baseline eGFR was positively correlated with OS after adjustment for the other factors in all patients. This was different than what was reported before by Sidiqi *et al.*^7^ where baseline eGFR had no effect on OS after ASCT in AL amyloidosis patients. In addition, in our study, patients with higher eGFR at 6 months post-ASCT had significantly better OS and patients with higher eGFR at 2 years post-ASCT.

Several previous studies have investigated predictors of survival after ASCT in AL amyloidosis patients. Cardiac involvement has been indicated as a negative predictor of survival whereas achieving hematologic VGPR or CR and conditioning with high-dose melphalan (>200 mg/m^2^) have been associated with improved survival.^1,18–21^

There might be several explanations for the favorable survival outcomes in patients with higher eGFR due to direct and indirect mechanisms. One possible reason is that patients with higher eGFR at baseline might be presenting with less organ involvement by amyloid and less hematologic disease burdens at the time of diagnosis. For example, cardiac amyloidosis can affect eGFR through the mechanisms of cardiorenal syndrome. Therefore, some of the patients with lower eGFR at baseline might be having lower eGFR due to cardiorenal syndrome caused by cardiac amyloidosis, and cardiac involvement is a known predictor of worse survival in patients with AL amyloidosis.

Moreover, the association of lower eGFR with poor survival outcomes might be due to indirect relationship of eGFR with higher hematologic disease burden.

In our study, higher serum-free kappa and lambda chain levels were associated with lower eGFR, whereas higher serum hemoglobin level was associated with higher eGFR at all time points.

On the other hand, eGFR is also a direct marker for the extent of amyloid deposition in the kidneys. After using Palladini's kidney amyloidosis staging, we found that those who were stage 3 at baseline (eGFR <50 ml/min per 1.73 m^2^, proteinuria >5 g/24 h) had inferior OS than those who were stage 1 (eGFR >50 ml/min per 1.73 m^2^, proteinuria <5 g/24 h). This finding suggests direct effect of kidney amyloid deposition on survival. However, our finding regarding advanced stage of kidney amyloidosis at baseline correlating with worse OS is different than what Palladini *et al.*^2^ and Havasi *et al.*^22^ have published before. Both Palladini and Havasi showed that higher baseline kidney amyloidosis stage defined with lower eGFR and higher proteinuria criteria predicted worse kidney outcome such as progression to dialysis but was not significantly associated with OS. This difference might be due to the lack of the statistical power due to small number of patients with kidney involvement in our study. A more recent multicenter study which evaluated kidney light chain amyloidosis using four different kidney response criteria concluded that patients that achieved kidney response by 24 months had a better OS compared with patients that had no kidney response.^23^

Several studies showed improvement of kidney function after ASCT at long-term follow-up.^24^ In our study, 108 patients had kidney involvement of AL amyloidosis and 55% had complete kidney response within 6 months after ASCT. The organ response in AL amyloidosis lags behind hematologic response, and it has been reported that it may take up to 12–24 months to see kidney response after ASCT.^25,26^ Owing to lack of kidney response data in our study at time points after 6 months post-ASCT, our study is unable to address a definitive conclusion about the effect of ASCT on kidney response. However, we observed that early kidney response within 6 months of ASCT was associated with improved OS.

Another interesting finding of our study is that patients with kidney progression at 6 months post-ASCT had inferior OS compared with those who had complete kidney response irrespective of the baseline stage of kidney amyloidosis. The lack of correlation between advanced stage of kidney amyloidosis at baseline and kidney disease progression, regarding the effect on survival, suggests that nonamyloid kidney complications of ASCT might be contributing to kidney progression at 6 months. AKI has been reported with an incidence of 21%–29% after ASCT in AL amyloidosis patients, and such high incidence seems to be due to infections, nephrotoxic medications, thrombotic microangiopathy, and other complications.^26,27^ Therefore, some of the patients meeting kidney progression criteria (≥25% decrease in eGFR at a given time point relative to baseline) in our study might have eGFR reduction due to episodes of AKI after ASCT rather than effects of AL amyloid deposition in the kidneys. However, at this point, we are not able to come to this conclusion objectively as we have not analyzed data regarding episodes of AKI in our cohort. Regardless of the reason for eGFR reduction, the effect on OS was not favorable in our study.

Our study has several limitations. First, given the retrospective nature of the study, some important data were missing. Owing to missing data regarding proteinuria, we were not able to assess association of kidney response with long-term outcomes comprehensively. In addition, we did not have a control group to compare kidney and survival outcomes for AL amyloidosis patients undergoing melphalan and ASCT vs other types of treatments. The number of patients with kidney involvement was only one third of the whole cohort in our study limiting analysis of the effect of kidney amyloidosis staging and kidney response on survival.

Our strength includes a relatively large cohort and that we evaluated factors associated with eGFR after ASCT in AL amyloidosis patients and further focused on survival outcomes of patients with kidney amyloidosis using both kidney staging and kidney response criteria.

This multicenter large-scale retrospective study demonstrates that kidney function has an effect on survival in patients with AL amyloidosis who undergo ASCT. Patients with higher eGFR and lower kidney amyloidosis stage at baseline, as well as patients who achieve complete kidney response at 6 months after ASCT, have favorable survival outcomes.

## Supplementary Material

SUPPLEMENTARY MATERIAL

## Data Availability

All original data, including deidentified patient-level data or individual laboratory data measurements, are included in the manuscript and/or supplemental material.

## Declarative Statements

This study includes clinical experimentation and received Institutional Review Board or Ethics Committee approval. The need to obtain informed patient consent was waived. This study includes clinical experimentation and complies with the Declaration of Helsinki.

## Supplemental Material

Supplemental Table 1. Staging of kidney AL amyloidosis.

Supplemental Table 2. Criteria of kidney response.

Supplemental Table 3A. Associations of PFS with patients' clinical, tumor characteristics, and baseline laboratory measures in multivariable setting at baseline, 100 days, and 6 months.

Supplemental Table 3B. Associations of PFS with patients' clinical, tumor characteristics, and baseline laboratory measures in multivariable setting at 1 year, 2 years, and 3 years.

Supplemental Table 4A. Associations of OS with patients' clinical, tumor characteristics, and laboratory measures at baseline, 100 days, and 6 months in multivariable settings.

Supplemental Table 4B. Associations of OS with patients' clinical, tumor characteristics, and laboratory measures at 1 year, 2 years, and 3 years in multivariable settings.
