# Supplementary material for: The Association of Kidney Function on Survival in Patients with Amyloid Light Chain Amyloidosis after Autologous Stem Cell Transplantation: A Multicenter Study
Source: Kidney360. 2025 Nov 10;7(3):568–82. doi: 10.34067/KID.0000001036 (PMC13065126; doi:10.34067/KID.0000001036)
Supplement: SUPPLEMENTARY MATERIAL [file kidney360-7-568-s001.pdf]

ASN American Society of Nephrology
JASN Journal of the American Society of Nephrology
KIDNEY360 Accessing Our World From Every Angle
CJASN Clinical Journal of the American Society of Nephrology

# ASN Journal Disclosure Form

As per ASN journal policy, I have disclosed any financial relationships or commitments I have held in the past 36 months as included below. I have listed my Current Employer below to indicate there is a relationship requiring disclosure. If no relationship exists, my Current Employer is not listed.

A. Abudayyeh has nothing to disclose.

I understand that the information above will be published within the journal article, if accepted, and that failure to comply and/or to accurately and completely report the potential financial conflicts of interest could lead to the following: 1) Prior to publication, article rejection, or 2) Post-publication, sanctions ranging from, but not limited to, issuing a correction, reporting the inaccurate information to the authors' institution, banning authors from submitting work to ASN journals for varying lengths of time, and/or retraction of the published work.

Name: Ala Abudayyeh
Manuscript ID: K360-2025-000760R1
Manuscript Title: The Association of Kidney Function on Survival in Patients with AL Amyloidosis after Autologous Stem Cell Transplantation
Date of Completion: September 18, 2025
Disclosure Updated Date: September 18, 2025

ASN American Society of Nephrology | JASN Journal of the American Society of Nephrology | KIDNEY360 Accessing Our World From Every Angle | CJASN Clinical Journal of the American Society of Nephrology

# ASN Journal Disclosure Form

As per ASN journal policy, I have disclosed any financial relationships or commitments I have held in the past 36 months as included below. I have listed my Current Employer below to indicate there is a relationship requiring disclosure. If no relationship exists, my Current Employer is not listed.

G. Bianchi reports the following:
Consultancy: Prothena pharmaceuticals, consulting fees, personal; Pfizer, consulting fees, personal; Janssen, consulting fees, personal; Ownership Interest: Pfizer, stocks, $14,000, husband.; and Honoraria: Prothena pharmaceuticals, consulting fees, personal; Pfizer, consulting fees, personal; Janssen, consulting fees, personal.

I understand that the information above will be published within the journal article, if accepted, and that failure to comply and/or to accurately and completely report the potential financial conflicts of interest could lead to the following: 1) Prior to publication, article rejection, or 2) Post-publication, sanctions ranging from, but not limited to, issuing a correction, reporting the inaccurate information to the authors' institution, banning authors from submitting work to ASN journals for varying lengths of time, and/or retraction of the published work.

Name: Giada Bianchi
Manuscript ID: K360-2025-000760R1
Manuscript Title: The Association of Kidney Function on Survival in Patients with AL Amyloidosis after Autologous Stem Cell Transplantation
Date of Completion: September 22, 2025
Disclosure Updated Date: September 22, 2025

ASN American Society of Nephrology | JASN Journal of the American Society of Nephrology | KIDNEY360 Accessing Our World From Every Angle | CJASN Clinical Journal of the American Society of Nephrology

# ASN Journal Disclosure Form

As per ASN journal policy, I have disclosed any financial relationships or commitments I have held in the past 36 months as included below. I have listed my Current Employer below to indicate there is a relationship requiring disclosure. If no relationship exists, my Current Employer is not listed.

R. Delgado reports the following:
Employer: MD Anderson Cancer Center

I understand that the information above will be published within the journal article, if accepted, and that failure to comply and/or to accurately and completely report the potential financial conflicts of interest could lead to the following: 1) Prior to publication, article rejection, or 2) Post-publication, sanctions ranging from, but not limited to, issuing a correction, reporting the inaccurate information to the authors' institution, banning authors from submitting work to ASN journals for varying lengths of time, and/or retraction of the published work.

Name: Ruby E Delgado
Manuscript ID: K360-2025-000760R1
Manuscript Title: The Association of Kidney Function on Survival in Patients with AL Amyloidosis after Autologous Stem Cell Transplantation
Date of Completion: September 22, 2025
Disclosure Updated Date: September 22, 2025

ASN American Society of Nephrology
JASN Journal of the American Society of Nephrology
KIDNEY360 Accessing Our World From Every Angle
CJASN Clinical Journal of the American Society of Nephrology

# ASN Journal Disclosure Form

As per ASN journal policy, I have disclosed any financial relationships or commitments I have held in the past 36 months as included below. I have listed my Current Employer below to indicate there is a relationship requiring disclosure. If no relationship exists, my Current Employer is not listed.

B. Dincer has nothing to disclose.

I understand that the information above will be published within the journal article, if accepted, and that failure to comply and/or to accurately and completely report the potential financial conflicts of interest could lead to the following: 1) Prior to publication, article rejection, or 2) Post-publication, sanctions ranging from, but not limited to, issuing a correction, reporting the inaccurate information to the authors' institution, banning authors from submitting work to ASN journals for varying lengths of time, and/or retraction of the published work.

Name: Berkay Ceyhun Dincer
Manuscript ID: K360-2025-000760R1
Manuscript Title: The Association of Kidney Function on Survival in Patients with AL Amyloidosis after Autologous Stem Cell Transplantation
Date of Completion: September 23, 2025
Disclosure Updated Date: September 23, 2025

ASN American Society of Nephrology
JASN Journal of the American Society of Nephrology
KIDNEY360 Accessing Our World From Every Angle
CJASN Clinical Journal of the American Society of Nephrology

# ASN Journal Disclosure Form

As per ASN journal policy, I have disclosed any financial relationships or commitments I have held in the past 36 months as included below. I have listed my Current Employer below to indicate there is a relationship requiring disclosure. If no relationship exists, my Current Employer is not listed.

K. Joachim reports the following:
Employer: University of California Los Angeles David Geffen School of Medicine

I understand that the information above will be published within the journal article, if accepted, and that failure to comply and/or to accurately and completely report the potential financial conflicts of interest could lead to the following: 1) Prior to publication, article rejection, or 2) Post-publication, sanctions ranging from, but not limited to, issuing a correction, reporting the inaccurate information to the authors' institution, banning authors from submitting work to ASN journals for varying lengths of time, and/or retraction of the published work.

Name: Kole P Joachim
Manuscript ID: K360-2025-000760R1
Manuscript Title: The Association of Kidney Function on Survival in Patients with AL Amyloidosis after Autologous Stem Cell Transplantation.
Date of Completion: September 23, 2025
Disclosure Updated Date: September 23, 2025

ASN American Society of Nephrology
JASN Journal of the American Society of Nephrology
KIDNEY360 Accessing Our World From Every Angle
CJASN Clinical Journal of the American Society of Nephrology

# ASN Journal Disclosure Form

As per ASN journal policy, I have disclosed any financial relationships or commitments I have held in the past 36 months as included below. I have listed my Current Employer below to indicate there is a relationship requiring disclosure. If no relationship exists, my Current Employer is not listed.

Y. Lin has nothing to disclose.

I understand that the information above will be published within the journal article, if accepted, and that failure to comply and/or to accurately and completely report the potential financial conflicts of interest could lead to the following: 1) Prior to publication, article rejection, or 2) Post-publication, sanctions ranging from, but not limited to, issuing a correction, reporting the inaccurate information to the authors' institution, banning authors from submitting work to ASN journals for varying lengths of time, and/or retraction of the published work.

Name: Yan Heather Lin
Manuscript ID: K360-2025-000760R1
Manuscript Title: The Association of Kidney Function on Survival in Patients with AL Amyloidosis after Autologous Stem Cell Transplantation
Date of Completion: September 23, 2025
Disclosure Updated Date: September 23, 2025

ASN American Society of Nephrology | JASN Journal of the American Society of Nephrology | KIDNEY360 Accessing Our World From Every Angle | CJASN Clinical Journal of the American Society of Nephrology

# ASN Journal Disclosure Form

As per ASN journal policy, I have disclosed any financial relationships or commitments I have held in the past 36 months as included below. I have listed my Current Employer below to indicate there is a relationship requiring disclosure. If no relationship exists, my Current Employer is not listed.

C. Martinez reports the following:
Employer: MD Anderson Cancer Center; Ownership Interest: Many to list but none related to the paper; and Advisory or Leadership Role: Informatics Chair American Society or Transplant and Cellular Therapy - ASTCT.

I understand that the information above will be published within the journal article, if accepted, and that failure to comply and/or to accurately and completely report the potential financial conflicts of interest could lead to the following: 1) Prior to publication, article rejection, or 2) Post-publication, sanctions ranging from, but not limited to, issuing a correction, reporting the inaccurate information to the authors' institution, banning authors from submitting work to ASN journals for varying lengths of time, and/or retraction of the published work.

Name: Charles Martinez
Manuscript ID: K360-2025-000760R1
Manuscript Title: The Association of Kidney Function on Survival in Patients with AL Amyloidosis after Autologous Stem Cell Transplantation
Date of Completion: September 24, 2025
Disclosure Updated Date: September 24, 2025

ASN
American Society of Nephrology
JASN
Journal of the American Society of Nephrology
KIDNEY360
Accessing Our World From Every Angle
CJASN
Clinical Journal of the American Society of Nephrology

# ASN Journal Disclosure Form

As per ASN journal policy, I have disclosed any financial relationships or commitments I have held in the past 36 months as included below. I have listed my Current Employer below to indicate there is a relationship requiring disclosure. If no relationship exists, my Current Employer is not listed.

S. Motwani reports the following:
Employer: Brigham and Women's Hospital and Dana-Farber Cancer Institute

I understand that the information above will be published within the journal article, if accepted, and that failure to comply and/or to accurately and completely report the potential financial conflicts of interest could lead to the following: 1) Prior to publication, article rejection, or 2) Post-publication, sanctions ranging from, but not limited to, issuing a correction, reporting the inaccurate information to the authors' institution, banning authors from submitting work to ASN journals for varying lengths of time, and/or retraction of the published work.

Name: Shveta S. Motwani
Manuscript ID: K360-2025-000760R1
Manuscript Title: The Association of Kidney Function on Survival in Patients with AL Amyloidosis after Autologous Stem Cell Transplantation
Date of Completion: September 23, 2025
Disclosure Updated Date: September 23, 2025

ASN American Society of Nephrology | JASN Journal of the American Society of Nephrology | KIDNEY360 Accessing Our World From Every Angle | CJASN Clinical Journal of the American Society of Nephrology

# ASN Journal Disclosure Form

As per ASN journal policy, I have disclosed any financial relationships or commitments I have held in the past 36 months as included below. I have listed my Current Employer below to indicate there is a relationship requiring disclosure. If no relationship exists, my Current Employer is not listed.

N. Murakami reports the following:
Employer: Washington University in St. Louis; Consultancy: Otsuka Pharmaceuticals; Asahi Kasei Inc.; Research Funding: Regeneron; Honoraria: Otsuka Inc.; Sanofi; and Speakers Bureau: Otsuka Inc.

I understand that the information above will be published within the journal article, if accepted, and that failure to comply and/or to accurately and completely report the potential financial conflicts of interest could lead to the following: 1) Prior to publication, article rejection, or 2) Post-publication, sanctions ranging from, but not limited to, issuing a correction, reporting the inaccurate information to the authors' institution, banning authors from submitting work to ASN journals for varying lengths of time, and/or retraction of the published work.

Name: Naoka Murakami
Manuscript ID: K360-2025-000760R1
Manuscript Title: The Association of Kidney Function on Survival in Patients with AL Amyloidosis after Autologous Stem Cell Transplantation
Date of Completion: September 19, 2025
Disclosure Updated Date: September 19, 2025

ASN American Society of Nephrology JASN Journal of the American Society of Nephrology KIDNEY360 Accessing Our World From Every Angle CJASN Clinical Journal of the American Society of Nephrology

# ASN Journal Disclosure Form

As per ASN journal policy, I have disclosed any financial relationships or commitments I have held in the past 36 months as included below. I have listed my Current Employer below to indicate there is a relationship requiring disclosure. If no relationship exists, my Current Employer is not listed.

V. Page reports the following:
Employer: The UT M. D. Anderson Cancer Center

I understand that the information above will be published within the journal article, if accepted, and that failure to comply and/or to accurately and completely report the potential financial conflicts of interest could lead to the following: 1) Prior to publication, article rejection, or 2) Post-publication, sanctions ranging from, but not limited to, issuing a correction, reporting the inaccurate information to the authors' institution, banning authors from submitting work to ASN journals for varying lengths of time, and/or retraction of the published work.

Name: Valda D. Page
Manuscript ID: K360-2025-000760R1
Manuscript Title: The Association of Kidney Function on Survival in Patients with AL Amyloidosis after Autologous Stem Cell Transplantation
Date of Completion: September 22, 2025
Disclosure Updated Date: September 22, 2025

ASN American Society of Nephrology · JASN Journal of the American Society of Nephrology · KIDNEY360 Accessing Our World From Every Angle · CJASN Clinical Journal of the American Society of Nephrology

# ASN Journal Disclosure Form

As per ASN journal policy, I have disclosed any financial relationships or commitments I have held in the past 36 months as included below. I have listed my Current Employer below to indicate there is a relationship requiring disclosure. If no relationship exists, my Current Employer is not listed.

M. Qazilbash reports the following:
Employer: MD Anderson Cancer Center; Consultancy: Sanofi-Genzyme; and Research Funding: Sanofi-Genzyme; Johnson & Johnson.

I understand that the information above will be published within the journal article, if accepted, and that failure to comply and/or to accurately and completely report the potential financial conflicts of interest could lead to the following: 1) Prior to publication, article rejection, or 2) Post-publication, sanctions ranging from, but not limited to, issuing a correction, reporting the inaccurate information to the authors' institution, banning authors from submitting work to ASN journals for varying lengths of time, and/or retraction of the published work.

Name: Muzaffar Qazilbash
Manuscript ID: K360-2025-000760R1
Manuscript Title: The Association of Kidney Function on Survival in Patients with AL Amyloidosis after Autologous Stem Cell Transplantation
Date of Completion: September 23, 2025
Disclosure Updated Date: September 23, 2025

ASN American Society of Nephrology
JASN Journal of the American Society of Nephrology
KIDNEY360 Accessing Our World From Every Angle
CJASN Clinical Journal of the American Society of Nephrology

# ASN Journal Disclosure Form

As per ASN journal policy, I have disclosed any financial relationships or commitments I have held in the past 36 months as included below. I have listed my Current Employer below to indicate there is a relationship requiring disclosure. If no relationship exists, my Current Employer is not listed.

G. Rondon reports the following:
Employer: MDAnderson Cancer Center

I understand that the information above will be published within the journal article, if accepted, and that failure to comply and/or to accurately and completely report the potential financial conflicts of interest could lead to the following: 1) Prior to publication, article rejection, or 2) Post-publication, sanctions ranging from, but not limited to, issuing a correction, reporting the inaccurate information to the authors' institution, banning authors from submitting work to ASN journals for varying lengths of time, and/or retraction of the published work.

Name: Gabriela Rondon
Manuscript ID: K360-2025-000760R1
Manuscript Title: The Association of Kidney Function on Survival in Patients with AL Amyloidosis after Autologous Stem Cell Transplantation
Date of Completion: October 21, 2025
Disclosure Updated Date: October 21, 2025

ASN American Society of Nephrology
JASN Journal of the American Society of Nephrology
KIDNEY360 Accessing Our World From Every Angle
CJASN Clinical Journal of the American Society of Nephrology

# ASN Journal Disclosure Form

As per ASN journal policy, I have disclosed any financial relationships or commitments I have held in the past 36 months as included below. I have listed my Current Employer below to indicate there is a relationship requiring disclosure. If no relationship exists, my Current Employer is not listed.

U. Selamet reports the following:
Employer: Dana Farber Cancer Center, Boston Medical Center; Consultancy: Mercor; and Ownership Interest: Nvidia, grail.

I understand that the information above will be published within the journal article, if accepted, and that failure to comply and/or to accurately and completely report the potential financial conflicts of interest could lead to the following: 1) Prior to publication, article rejection, or 2) Post-publication, sanctions ranging from, but not limited to, issuing a correction, reporting the inaccurate information to the authors' institution, banning authors from submitting work to ASN journals for varying lengths of time, and/or retraction of the published work.

Name: Umut Selamet
Manuscript ID: K360-2025-000760R1
Manuscript Title: The Association of Kidney Function on Survival in Patients with AL Amyloidosis after Autologous Stem Cell Transplantation
Date of Completion: September 22, 2025
Disclosure Updated Date: September 22, 2025
