# Supplementary material for: The Association of Kidney Function on Survival in Patients with Amyloid Light Chain Amyloidosis after Autologous Stem Cell Transplantation: A Multicenter Study
Source: Kidney360. 2025 Nov 10;7(3):568–82. doi: 10.34067/KID.0000001036 (PMC13065126; doi:10.34067/KID.0000001036)
Supplement: SUPPLEMENTARY MATERIAL [file kidney360-7-568-s002.pdf]

Supplemental Tables:

Supplemental Table 1. Staging of kidney AL amyloidosis	
Markers and thresholds	Stages
eGFR <50 mL/min per 1.73 m^2<br>proteinuria >5 g/24h	I. Both eGFR above and proteinuria below the cutoffs<br>II. Either eGFR below or proteinuria above the cutoffs<br>III. Both eGFR below and proteinuria above the cutoffs

**Supplemental Table 2. Criteria of kidney response**	
**Markers and thresholds**	**Response**
≥25% decrease in eGFR relative to baseline	Progression
<25% decrease in eGFR relative to baseline and proteinuria ≤ 200mg/24h at 6 months post-transplant	Complete response
<25% decrease in eGFR relative to baseline and proteinuria >60% reduction at 6 months post-transplant relative to baseline	Very good partial response
<25% decrease in eGFR relative to baseline and proteinuria 31-60% reduction at 6 months post-transplant relative to baseline	Partial response
<25% decrease in eGFR relative to baseline and proteinuria ≤30% reduction at 6 months post-transplant relative to baseline	No response

Supplemental Table 3a. Associations of PFS with patients' clinical, tumor characteristics and baseline lab measures in multivariable setting at baseline, 100 days, and 6 months

		Baseline			100 days			6 months		
Parameter	Level	HR (95% CI)	p*	p**	HR (95% CI)	p*	p**	HR (95% CI)	p*	p**
Kidney Amyloidosis	Yes vs No	0.665 (0.457, 0.969)	0.0335	0.0335	0.564 (0.374, 0.851)	0.0064	0.0064	0.556 (0.362, 0.854)	0.0074	0.0074
Melphalan Dose	≥200 mg/m^2 vs <200 mg/m^2	0.694 (0.492, 0.979)	0.0375	0.0375	0.662 (0.46, 0.953)	0.0264	0.0264	0.579 (0.391, 0.859)	0.0066	0.0066
eGFR	Q4 vs Q1	1.028 (0.619, 1.708)	0.9152	0.9540	0.953 (0.559, 1.624)	0.8581	0.2976	1.203 (0.64, 2.262)	0.5661	0.8499
	Q3 vs Q1	0.926 (0.569, 1.506)	0.7554		0.673 (0.402, 1.125)	0.1306		0.929 (0.531, 1.624)	0.7957	
	Q2 vs Q1	1.052 (0.683, 1.621)	0.8165		1.068 (0.682, 1.673)	0.7739		1.057 (0.63, 1.772)	0.8337	
Kappa/Lambda	Q4 vs Q1	2.693 (1.591, 4.559)	0.0002	0.0015	1.881 (1.12, 3.158)	0.0169	0.0588	1.897 (1.008, 3.57)	0.0473	0.0750
	Q3 vs Q1	2.178 (1.303, 3.64)	0.0030		1.662 (0.994, 2.777)	0.0527		1.806 (0.998, 3.267)	0.0508	
	Q2 vs Q1	1.538 (0.907, 2.608)	0.1103		1.174 (0.694, 1.987)	0.5494		1.121 (0.631, 1.991)	0.6968	

*p value for comparison with reference group
**p value for overall effect
HR: hazard ratio
CI: confidence interval

Supplemental Table 3b. Associations of PFS with patients' clinical, tumor characteristics and baseline lab measures in multivariable setting at 1 year, 2 years and 3 years

		1 year			2 years			3 years		
Parameter	Level	HR (95% CI)	p*	p**	HR (95% CI)	p*	p**	HR (95% CI)	p*	p**
AMYLOIDOSIS_SITE__Ki	Yes vs No	0.515 (0.321, 0.828)	0.0061	0.0061						
Albumin	Q4 vs Q1							0.206 (0.043, 0.987)	0.0481	0.0458
	Q3 vs Q1							0.469 (0.157, 1.4)	0.1747	
	Q2 vs Q1							1.193 (0.467, 3.05)	0.7128	
eGFR	Q4 vs Q1	2.167 (1.013, 4.635)	0.0463	0.1363	1.989 (0.695, 5.695)	0.2001	0.4793	5.636 (1.512, 21.01)	0.0100	0.0372
	Q3 vs Q1	1.433 (0.696, 2.952)	0.3287		1.495 (0.575, 3.882)	0.4094		1.723 (0.499, 5.951)	0.3899	
	Q2 vs Q1	1.995 (0.981, 4.054)	0.0563		2.017 (0.774, 5.255)	0.1512		1.342 (0.442, 4.075)	0.6042	
Kappa/Lambda	Q4 vs Q1	3.853 (1.758, 8.446)	0.0008	0.0002	6.191 (2.007, 19.098)	0.0015	0.0025	20.24 (3.937, 104.045)	0.0003	0.0003
	Q3 vs Q1	3.241 (1.67, 6.29)	0.0005		5.218 (2.013, 13.525)	0.0007		21.145 (5.113, 87.447)	<.0001	
	Q2 vs Q1	1.348 (0.697, 2.607)	0.3746		2.37 (0.893, 6.292)	0.0831		11.507 (2.723, 48.62)	0.0009	

*p value for comparison with reference group
**p value for overall effect; HR: hazard ratio ;CI: confidence interval

Supplemental Table 4a. Associations of OS with patients' clinical, tumor characteristics and lab measures at baseline, 100 days and 6 months in multivariable settings

		Baseline			100 days			6 months		
Parameter	Level	HR (95% CI)	p*	p**	HR (95% CI)	p*	p**	HR (95% CI)	p*	p**
Age at Transplant	Per year increase	1.041 (1.019, 1.063)	0.0003	0.0003	1.046 (1.02, 1.073)	0.0004	0.0004	1.047 (1.022, 1.073)	0.0002	0.0002
Kidney Amyloidosis	Yes vs No									
Melphalan Dose	≥200 vs <200							0.578 (0.356, 0.938)	0.0266	0.0266
eGFR	Q4 vs Q1	0.403 (0.236, 0.688)	0.0009	0.0002	0.491 (0.269, 0.898)	0.0209	0.0025	0.585 (0.294, 1.164)	0.1266	0.0242
	Q3 vs Q1	0.368 (0.214, 0.633)	0.0003		0.339 (0.171, 0.672)	0.0019		0.527 (0.268, 1.037)	0.0635	
	Q2 vs Q1	0.806 (0.512, 1.27)	0.3529		0.939 (0.561, 1.572)	0.8120		1.241 (0.718, 2.145)	0.4388	
Kappa/Lambda	Q4 vs Q1				1.658 (0.924, 2.975)	0.0901	0.0375	1.933 (0.961, 3.886)	0.0644	0.0378
	Q3 vs Q1				1.256 (0.686, 2.297)	0.4602		1.223 (0.599, 2.498)	0.5798	
	Q2 vs Q1				0.651 (0.322, 1.316)	0.2316		0.852 (0.42, 1.726)	0.6561	

*p value for comparison with reference group
**p value for overall effect
HR: hazard ratio
CI: confidence interval

Supplemental Table 4b. Associations of OS with patients' clinical, tumor characteristics and lab measures at 1 year, 2 years and 3 years in multivariable settings

		1 year			2 years			3 years		
Parameter	Level	HR (95% CI)	p*	p**	HR (95% CI)	p*	p**	HR (95% CI)	p*	p**
Age at Transplant	Per year increase	1.045 (1.017, 1.073)	0.0013	0.0013	1.065 (1.026, 1.105)	0.0008	0.0008			
Kidney Amyloidosis	Yes vs No	0.538 (0.307, 0.944)	0.0307	0.0307				0.295 (0.133, 0.657)	0.0028	0.0028
Melphalan Dose	≥200 vs <200	0.584 (0.34, 1.003)	0.0514	0.0514						
eGFR	Q4 vs Q1	1.629 (0.697, 3.807)	0.2599	0.0727	1.265 (0.527, 3.039)	0.5992	0.0430	1.014 (0.349, 2.944)	0.9796	0.3147
	Q3 vs Q1	0.824 (0.373, 1.823)	0.6331		0.276 (0.07, 1.084)	0.0651		0.386 (0.127, 1.167)	0.0918	
	Q2 vs Q1	1.78 (0.97, 3.264)	0.0625		1.591 (0.664, 3.814)	0.2975		0.714 (0.303, 1.68)	0.4402	
Kappa-Lambda	Q4 vs Q1	4.759 (1.974, 11.474)	0.0005	0.0001	13.039 (3.536, 48.08)	0.0001	0.0006			
	Q3 vs Q1	1.659 (0.684, 4.02)	0.2628		4.32 (1.208, 15.45)	0.0244				
	Q2 vs Q1	1.277 (0.519, 3.142)	0.5952		4.081 (1.07, 15.561)	0.0395				
Hemoglobin	Q4 vs Q1	0.538 (0.232, 1.25)	0.1498	0.0462				0.17 (0.057, 0.508)	0.0015	0.0012
	Q3 vs Q1	0.372 (0.187, 0.743)	0.0051					0.152 (0.045, 0.511)	0.0023	
	Q2 vs Q1	0.687 (0.389, 1.213)	0.1952					0.283 (0.119, 0.674)	0.0043	

*p value for comparison with reference group
**p value for overall effect
HR: hazard ratio
CI: confidence interval
